# Traditional medicinal plants used in the treatment of tuberculosis in Ethiopia: A systematic review

**DOI:** 10.1016/j.heliyon.2022.e09478

**Published:** 2022-05-18

**Authors:** Samuel Getachew, Girmay Medhin, Abyot Asres, Gemeda Abebe, Gobena Ameni

**Affiliations:** a Department of Biology, College of Natural and Computational Sciences, Mizan-Tepi University, PO Box 121, Tepi, Ethiopia; b Aklilu Lemma Institute of Pathobiology, Addis Ababa University, PO Box 1176, Addis Ababa, Ethiopia; c School of Public Health, College of Health Sciences, Mizan Tepi University, Mizan-Aman, Ethiopia; d School of Medical Laboratory Science and Mycobacteriology Research Center, Jimma University, Jimma, Ethiopia; e Department of Veterinary Medicine, College of Agriculture and Veterinary Medicine, United Arab Emirates University, PO Box 15551, Al Ain, United Arab Emirates

**Keywords:** Medicinal plants, Traditional, Treatments, Tuberculosis, Ethiopia

## Abstract

**Background:**

Majority of people in Ethiopia heavily rely on traditional medicinal plants to treat a number of diseases including tuberculosis (TB). However, there has been lack of comprehensive evidences on taxonomic distribution of medicinal plant species, methods of preparation of remedies from these plants and how the remedies are administered. This systematic review is designed to examine and synthesize available evidences focusing on medicinal plants that have been used for TB treatment in Ethiopia.

**Methods:**

Research findings related to ethno-botanical and pharmacological approaches of TB remedies were retrieved from databases. Electronic libraries of Ethiopian Universities and relevant church-based religious books were also reviewed as additional sources. Evidences are searched and organized in accordance with the Preferred Reporting Items for Systematic Reviews and Meta-Analyses (PRISMA) guideline.

**Result:**

From a total of 68 research documents that reported use of plants for treatment of TB 98 plants species belonging to 82 genera and 49 families were identified. The most frequently reported plant species belonged to family *Lamiaceae* (n = 8), *Euphorbiaceae* (n = 7), *Cucurbitaceae* (n = 6) and *Fabaceae* (n = 6). *Croton macrostachyus, Allium sativum, and Myrsine Africana* were the most often mentioned anti-TB medicinal plants. Shrubs (35.7%) and trees (29.6%) were reported as dominant growth forms while plant roots (31.6%) and leaves (28.6%) were frequently used plant parts for the preparations of the treatment. The most favored administration route was oral (59.1%). About 87% of the preparations were made from fresh plant materials. No experimental/clinical evidence was presented for 79.6%(78/98) of the reported plants to support their anti-mycobacterial activities.

**Conclusion:**

In Ethiopia, the number of herbal remedies is enormous and their use for TB treatment is a common practice. However, majority of them are not yet backed up by evidence generated through scientific experimentation and this warrants further experimental and clinical validations. Moreover, the efficacy, toxicity and safety tests should be initiated and this would help in the rapid identification of new anti-TB regimens, and possibly it would lead to developing more effective new plant-based drugs. This systematic review will serve as a reference for the selection of plants for developing new anti-TB regimens.

## 1 Introduction

The current modern treatment of TB depends on rifampicin, ethambutol, isoniazid and pyrazinamide, which are less effective (Brigden et al., 2014) and costly with serious side-effects (Bhatcha, 2013; Zazueta-Beltran et al., 2011; Mohan and Sharma, 2004). An emergence of drug resistant (Gupta et al., 2010; Zazueta-Beltran et al., 2011) and geographically specific strains of TB etiologies (Firdessa et al., 2013) has further exacerbated the situation (threat) in TB-burdened developing countries of Africa, and have necessitated a need to search for new treatment regimens that target medicinal plants (Andualem et al., 2014; Hostettmann et al., 2000; Kloos et al., 1978; Kloos, 1976; Askun et al., 2013; Bhatcha, 2013).

The use of medicinal plants remains the primary source of healthcare for majority of people in most of developing countries, it may reach 70–80% among the Africans, and it could be as high as 85% in the sub-Saharan Africa (Mann et al., 2008c; Mann et al., 2007; WHO, 2002; Zuberi, 2014; Andarge et al., 2015; Abbink, 2002; Obakiro et al., 2020). Medicinal plants may offer a new hope for developing alternative medicines for a number of diseases as they are easily accessible (Zuberi, 2014; Heinrich, 2000) and cheap with a minimum of side effects (Hostettmann et al., 2000; Siddiqui et al., 2014; Abebe, 1996). Plant derived medicines may also help in fighting drug resistance (Bhatcha, 2013; Singh et al., 2015) and combating geographically specific strains of TB etiologies (Gupta et al., 2010). Therefore, effective and alternative anti-TB drugs preferably plant-based ones have to be developed to fight drug resistance and to reduce TB associated mortality and morbidity (Andualem et al., 2014; Amsalu, 2010; Hostettmann et al., 2000; Enyew et al., 2014; Bishaw, 1991; Gupta et al., 2010).

In Ethiopia there are more than 6,600 vascular plant species (Bekele-Tesemma, 2007). From 70-80% of the Ethiopians still rely on traditional medicinal plants (TMPs) to treat a variety of diseases such as gastrointestinal (Belayneh et al., 2012; Bekalo et al., 2009), respiratory tract and sexually transmitted infections (Abera, 2014; Kewessa et al., 2015), hemorrhoids, rabies (Tesfahuneygn and Gebreegziabher, 2019), hypertension, diabetes (Andarge et al., 2015), malaria (Abbink, 2002; Alemneh, 2021a, Alemneh, 2021b; Agize et al., 2013) and others (FMOH, 2003; Negussie, 1988; Birhan et al., 2011). However, there has been no study that has synthesized existing evidence focusing on documentation of traditional medicinal plants (TMPs) being used in treating TB in Ethiopia. And this has resulted in unavailability of comprehensive data on plant species, methods of preparation and administration of traditional TB remedies. This systematic review was designed to address this gap by documenting existing TMPs that are being used in TB treatments in Ethiopia. In this paper we report synthesis of existing evidence that was obtained from a systematic review of the available literatures on anti-mycobacterial plants with the hope of providing comprehensive data to hasten the research effort on development of novel plant derived drugs against human and bovine TB.

## 2 Methods

This systematic review and analysis of peer reviewed journal articles, Msc/PhD theses/dissertations, and unpublished documents related to medicinal plants used for the treatment of TB [n = 68] in Ethiopia was conducted over nine month period from November 2020 to July 2021.

### 2.1 Literature search strategy

Web-based systematic search strategy was employed. Ethno-botanical/ethno-medicinal studies reporting on medicinal plants used for traditional TB treatment in Ethiopia were gathered through two different search modalities for published and unpublished research findings. Google search engine and local university websites were assessed for unpublished MSc/PhD thesis research reports while international scientific databases that include PubMed, Research gate, Science direct, Web of Science, Google Scholar, academia edu, and AJOL were used as sources of published journal articles. The search was done using several key terms: Ethiopia/Ethiopian plants/Ethiopian medicinal plants/anti-tuberculosis plants, anti-lymphadenitis/gland TB plants, traditional knowledge/TMPs, herbal medicine/remedies, indigenous knowledge, folk medicine/remedies, ethno-botany/ethno-botanical, ethno-pharmacological/medicine/, ethno-pharmaceutical, cultural medicine following “Preferred Reporting Items for Systematic Reviews and Meta-Analyses (PRISMA)” guidelines and guidance (Moher et al., 2009a, Moher et al., 2009b).

### 2.2 Inclusion criteria

Published and unpublished ethno-botanical/medicinal reports including experimental studies about treatments of TB in Ethiopia and reported before May 2021 were included.

### 2.3 Exclusion criteria

Information from published and unpublished ethno-botanical and ethno-medicinal surveys lacking scientific plant names and not reporting information about anti-TB medicinal plants were excluded from the analysis.

### 2.4 Screening and criteria

For this systematic review, the title and abstract of identified journal articles/theses/dissertations/reports were downloaded and all those suitable for the purpose were screened out and critically inspected for inclusion.

### 2.5 Data retrieval

A data collection tool was developed in Microsoft Excel format into which all retrieved data (botanical name, plant family, local name(s), part(s) used, habit of growth, preparation and administration mode, extraction method of each plant used for TB treatment), were entered. Missed information in some studies, particularly local name and habit of the plants, geographic locations of the study localities/districts, and misspelled scientific names were retrieved and corrected through direct web-searching.

### 2.6 Data analysis

All retrieved relevant data about the Ethiopian TMPs were entered into structured Microsoft office Excel format and exported to Statistical Software Packages for Social Science (SPSS, software version 20.0). Descriptive statistical methods, percentage and frequency were used to analyze ethno-botanical data on reported medicinal plants.

## 3 Results

**Figure 1:**
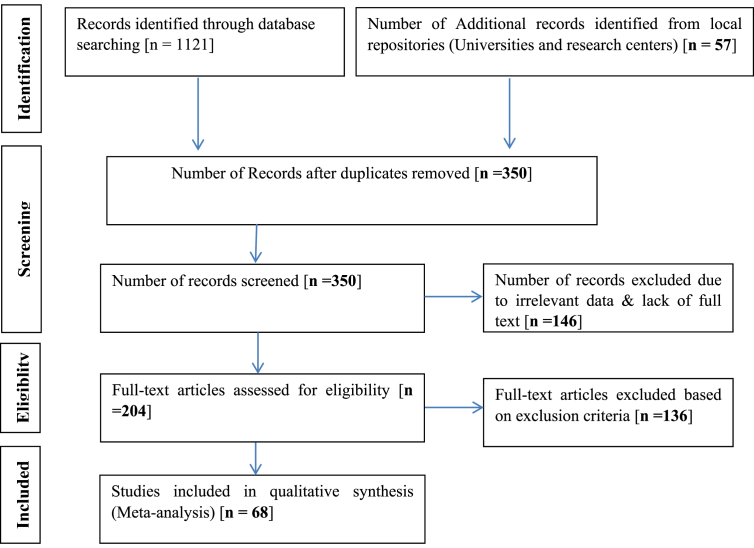
Flow chart of retrieved and analyzed literatures/papers (adapted from PRISMA, 2020) (Page et al., 2021).

Peer reviewed journal articles, M.Sc./Ph.D. theses/dissertations research reports representing ten different regional states of Ethiopia and other unpublished documents [n = 68] were included and analyzed in this review (Figure 1).

### 3.1 Taxonomic distribution of herbal medicines of TB in Ethiopia

A total of 98 different plant species that are used to treat TB traditionally were retrieved from 68 ethno-medicinal study reports recruited for this review. The plants were from 82 genera and 49 families. While taxonomic summary of reported plants is put in Table 1, detailed taxonomic and geographic distribution, habit, parts used, modes of preparation and routes of administration and dosage of herbal remedies of TB is found in Table 2.

**Table 1:** Taxonomic distribution of herbal medicines used for the treatment of TB in Ethiopia.

Family	Genera	Species	Family	Genera	Species
Lamiaceae	5	8	Anacardiaceae	2	2
Cucurbitaceae	4	6	Asclepiadaceae	2	2
Fabaceae	4	6	Combretaceae	2	2
Euphorbiaceae	5	7	Meliaceae	2	2
Asteraceae	3	3	Myrsinaceae	2	2
Capparidaceae	3	3	Rosaceae	2	2
Malvaceae	3	3	Rutaceae	2	2
Apocynaceae	2	3	Rubiaceae	2	2
Myrtaceae	2	3	Alliaceae	1	3
Oleaceae	2	3	Ranunculaceae	1	2
Solanaceae	2	3	Other families	29	29
**Total**				**82**	**98**

**Table 2:** Taxonomic and geographic distribution, habit, parts used, modes of preparation and routes of administration and dosage of herbal remedies of TB.

SN	Family Name	Botanical name	Common name(s)/language name/s	Region	Habit	Part used	ROA	Mode of preparation/Types of TB	References
1	Lamiaceae	*Artemisia abyssinica* Shc.Bip.ex.A.Rich	Tiroo (Oro)	Oro	H	Lv	Or	Not specified	(Gemechu et al., 2013; Bekalo et al., 2009)
2		*Artemisia afra* Jacq. ex Willd	Chiqugn (Amh)	Oro	H	Lv	Or	Not specified	(Bekalo et al., 2009; Yineger et al., 2008)
*3*		*Clerodendrum myricoides* Hochst. Vatke	Aghio (kaficho)	Kaffa	Sh		Or	Not specified	(Abate, 1989)
*4*		*Ocimum americanum* L.	Zeka-keba (Amh)	SNNP	H	Fr		Not specified	(Bekalo et al., 2009)
*5*		*Ocimum basilicum* L.	Besobilla (Amh)	Amh	H	Sd		Not specified	(Gemechu et al., 2013)
*6*		*Ocimum lamiifolum* Hochst. ex Benth....	Demakessie (Oro)	Oro	T	Lv		Fresh leaves pounded and juice is drunk	(Gizachew et al., 2013; Mesfin et al., 2005; Getahun, 1976)
*7*		*Oenanthe procumbens* (H. Wolff) Norman	Bunkaka Hida (Or)	Amh	Sh	Lv	Or, Sk	Oral/skin EPTB	(Amsalu, 2010)
*8*		*Otostegia integrifolia* Benth	Tinjute (Amh)	Amh	Sh	Rt	Or, Ins	Fresh or dried leaf is used as fire fumigation	(Kahaliw, 2016; Enyew et al., 2014)
*9*	Euphorbiaceae	*Clutia abyssinica* Kaub. & Spach.	Yemar semat (G)	SNNP	Sh	Lv	Or	Infusion	(Teka et al., 2020)
*10*		*Croton macrostachyus* Hochst*.* ex Delile	Masincho (Si)	SNNP	T	Ba	Or	Boiling leaves of shoots in water and decanting the toxic water, & allowed to dry. Mixing dry fine powder with powder of spices & water, and giving about two syringes per day for a month	(Tefera and Kim, 2019; Kewessa et al., 2015; Gonfa et al., 2015; Balcha et al., 2014; Gemechu et al., 2013; Amsalu, 2010; Geyid et al., 2005)
*11*		*Euphorbia candelabrum* Ketshy	Kulkual (Amh)	Amh/Oro	T	Lq	Or	Dropping diluted in water (drinking)	(Bekele and Reddy, 2014; Mesfin et al., 2013)
*12*		*Euphorbia tirucali* L.	Kenchib (Amh)		T	Lq		Not specified	(Genene and Hazare, 2017)
*13*		*Euphorbia cryptospinosa* Bally	Aananno (Oro)	Oro	C	Rt	Or	Crushing internal part of the root with the roots of *Solanum incanum* & *Osyris quadripartita*, making s/n & adding honey then drinking as necessary when the patients become thirsty	(Fenetahun and Eshetu, 2017; Ashagre et al., 2016)
*14*		*Jatropha glauca* Vahl*.*	Qablis (Af)	Afar	Sh	Rt	Or, Ins	Making infusion of fresh root and administering intranasal and orally	(Seifu, 2004)
*15*		*Ricinus communis* L.	Qobbo	Oro	Sh	Lv	Or	Rubbing fresh warmed leaf with fine on the swelling	(Wolditsadik, 2018)
*16*	Cucurbitaceae	*Coccinia abyssinica* (Lam.) Cogn	Anchote (Oro)	Oro	H	Rt	Or	Cooking its root with leaves of *Croton macrostachyus* and eating with ‘injera’ for four days	(Birhanu et al., 2015; Dawit and Estifanos, 1991; Megersa et al., 2013; Getahun, 1985; Amare, 1973)
*17*		*Cucumis dipsaceus* Ehrenb*.*	Hafaflo (Tig)	Tig	C	Rt	Or	Not specified	(Zenebe et al., 2012)
*18*		*Cucumis ficifolius* A.Rich	Yemdir embouy (Amh)	SNNP/Amh/Tig	H	Fr	Or	Mixing its fruit with root of *Gnidia involucrata* and bulb of garlic, crushing and soaking it 7 days in local “Tella” and taking one cup for five days or powdered, mixed with water, drink	(Araya et al., 2015; Regassa, 2013; Gebeyehu, 2011)
*19*		*Cucumis pastulatus* L.	Qalfoon (Som)	Oro	C	RT	Or	Chewing the root or crushing the root, making s/n and drinking one coffee cup daily until cured	(Ashagre et al., 2016; Balemie et al., 2004)
*20*		*Momordica foetida* Schumach	Yubarrae	SNNP	C	Rt	Or	Crushed/pounded fresh/dry root mixed with *Allium sativum* bulb is taken orally before breakfast for three days.	(Mesfin et al., 2009)
*21*		*Zehneria scabra* (Linn. f.) Sond.	Haregresa (Amh)	Amh	H	St, Lv	Sk/To	Not specified	(Alemneh, 2021a, Alemneh, 2021b)
*22*	Fabaceae	*Acacia albida* Del*.*	Gerbi (Oro)	Oro/SNNP	T	AP	Or	Concoction, crushed	(Temam and Dillo, 2016; Belayneh et al., 2012)
*23*		*Acacia mellifera* (M. Vahl) Benth	Kontir grar (Amh)	Afar	Sh	Lv	Or	ETPB (fresh leaves consumption)	(Teklehaymanot, 2017)
*24*		*Acacia oerfota* (Forssk.) Schweinf	Wanga (Or)	Afar	Sh	Rt	Or, Ins	Fresh root consumption	(Teklehaymanot, 2017)
*25*		*Calpurnia aurea* (Aiton) Benth.	Hitsawutse (Tig)	Tig	Sh	Rt	Or	Not specified	(Gemechu et al., 2013; Zenebe et al., 2012)
*26*		*Erythrina brucei* Schweinf	Woleko (Sid)	SNNP	T	Ba	Or	Not specified (Bovine TB)	(Kewessa et al., 2015)
*27*		*Pterolobium stellatum* (Forsk.) Brenan*.*	Kentefa (Amh)	Amh/Tig	Sh	Rt		Not specified	(Kahaliw, 2016; Balcha et al., 2014)
*28*	Alliaceae	*Allium cepa* L.	Qey shinkurt (Amh)	Oro	B	Bu	Or	Fresh chewing	(d’Avigdor et al., 2014; Fulas, 2007; Fullas, 2003)
*29*		*Allium ursinum* L.	Yejib shinkurt (Amh)	Tig	B	Fr	Or	Fresh fruits crushed & blended with honey & butter	(Balcha et al., 2014; Gemechu et al., 2013; Belayneh et al., 2012; Yirga, 2010)
*30*		*Allium sativum* L. H	Kashari shunkurutta (Oro)	Oro/SNNP/Tig	B	Bu/Lv	Or	Taking orally grinded and mixed with honey	(Osman et al., 2020; Belayneh et al., 2012; Mesfin et al., 2009; Wondimu et al., 2007)
*31*	Apocynaceae	*Carissa edulis* Vahl	Agam (Amh)	Amh	T	Rt	Or	Not specified	(Kahaliw, 2016)
*32*		*Carissa spinarum* L*.*	Otilaa (Si)	SNNP	Sh	Fr	Or	Not specified	(Kewessa et al., 2015)
*33*		*Kanahia laniflora* (Forssk.) R. Br.	Leehamohcaxa (Af)	Afar	Sh	Lv	Or, Ins	Making infusion of fresh leaves and administering intranasal and a small amount orally	(Seifu, 2004)
*34*	Asteraceae	*Echinops kebericho* Mesfin	kebericho (Oro)	Oro	H	Rt		Not specified	(d’Avigdor et al., 2014; Abebe et al., 2003)
*35*		*Laggera tomentosa* (Sch.Bip.ex A.Rich.) Oliv.& Hiern	Keskessie (Amh)	Amh	T	LV	Sk/To	Tying fresh pounded leaf on the swelling.	(Wolditsadik, 2018)
*36*		*Vernonia amygdalina* Del*.*	Grawa (Amh)	Amh	Sh	Rt		Not specified	(Kahaliw, 2016)
*37*	Capparidaceae	*Balanites rotundifolia* (van Tiegn) Blatter	Alayto (Af)	Afar	Sh	Lv	Or, Ins, Sk/To	Crsuhing leaves ETPB (Hu + Bovine TB)	(Teklehaymanot, 2017)
*38*		*Boscia angustifolia* A. Rich	Kermed (Tig)	Tig	T	Ba	Or	Crushing together with whole part of *Celtis Africana* homogenize with water and drinking a bottle cup of the solution for 7 consecutive days in the morning	(Gidey et al., 2015)
*39*		*Cadaba rotundifolia* Forssk	Kenquele (Kam)	Afar	Sh	Lv	Or, Ins	Bovine TB (fresh leaves consumption)	(Teklehaymanot, 2017)
*40*	Malvaceae	*Hibiscus cannabinus* L.	Dans's'a *(Dawro)*	SNNP	Sh	Fl	Or	Chopped, pound	(Agize et al., 2013)
*41*		*Malva parviflora* L	Siito (Halaba)	SNNP	H	Lv	Or	The leaf is crushed, powder mixed with water drunk	(Regassa et al., 2017)
*42*		*Sida schimperiana* Hochst. ex A. Rich	Chefreg (Amh)		H	Rt		Not specified	(Genene and Hazare, 2017)
*43*		*Eucalyptus spps.*	Bahir zaf (Amh)	Tig	T	Lv		Not specified	(Birhanu et al., 2015)
*44*	Myrtaceae	*Eucalyptus camaldulensis* Dehnh	Key bahir zaf (Amh)	Tig	T	Lv		Not specified	(Gemechu et al., 2013; Birhane et al., 2011)
*45*		*Syzygium guineense* (Willd.) DC.	Duwancho (Sid)	SNNP	T	Bk	Or	Not specified (used for both human and bovine TB)	(Kewessa et al., 2015)
*46*	Oleaceae	*Jasminum abyssinicum* Hochst*.*	Tembelel (Amh)	Amh	T	AP		Not specified	(Geyid et al., 2005)
*47*		*Olea europaea* L.	Woira (Amh)	Oro/SNNP/Afar	T	Fr	Or	Not specified	(Legesse et al., 2011; Teklehaymanot and Giday, 2010; Amenu, 2007)
*48*		*Olea europaea subsp. cuspidata* (Wall. Ex G.Don.) Cif	Ejersa (Oro)	Oro	T	Rt	Sk/To	The extracted oil from the roots put on the affected site (Bone TB) EPTB	(Jima and Megersa, 2018; Kewessa et al., 2015)
*49*	Solanaceae	*Capsicum annuum* L.	Geed case (Som)	Som	H	WP	Or	Grounding the stem and dissolving with water & drinking	(Issa, 2015)
*50*		*Solanum anguivai* Lam.	Ambu (Bench)	SNNP	Sh	Lv	Sk/To	Pounding leaf and apply topically for gland TB	(Giday, 2009a; 2009b)
*51*		*Solanum marginatum* L. f.	Abyiengule(Tig)	Tig	Sh	Sds	Or	Drying seeds, crushing & adding into milk or coffee and solution taking every morning for 21 days (even for cattle)	(Araya et al., 2015)
*52*	Anacardiaceae	*Rhus vulgaris* Meikle	Kammo (Amh)	Amh	Sh	Fr	Or	Grounding fruits are mixing with honey and one glass is drunk on empty stomach until recovery.	(Gebeyehu, 2011)
*53*		*Schinus Molle* L*.*	Kundo berbere (Amh)	Oro	T	Sd	Or	Crushing seeds and mixing with honey and eating	(Getaneh and Girma, 2013)
*54*	Asclepiadaceae	*Calotropis procera* (Ait.) Ait	Ginda (Tig)	Tig	Sh	Rt	Ins	Crushing its roots into powder and mix with pounded bark of *Croton macrostachyus* and leaves of *Ficus palmate* & sniffing	(Araya et al., 2015)
*55*		*Dregea sp.*	Geed sare (Sum)	Som	C	Lv	Or	Grinding leaves and boiling with milk and drinking	(Issa, 2015)
*56*	Combretaceae	*Combretum molle* G. Don	Xamasuda (Sum)	Som	T	Lv	Or	Grounding the leaves boiling and drinking	(Issa, 2015)
*57*		*Corrigiola capensis subsp. Africana*	Dakagella (ku) Kunama	Tig	T	Lv	Or	Crushing the leaf, and drink a cup of the juice for three consecutive days	(Gidey et al., 2015)
*58*	Meliaceae	*Trichilia dregeana* Sond	Anunu (Amh)	Oro	T	Rt	Or	Powdering and taking its 1/2 cup of tea	(Etana, 2015)
*59*		*Ekebergia capensis* Sparrm*.*	Olonchoo (Sid)	SNNP	T	Ba	Or	Crushing and pounding mixing with Hot Water/Bovine TB	(Tefera and Kim, 2019; Kewessa et al., 2015; Banerjee et al., 2014)
*60*	Myrsinaceae	*Embelia schimperi* Vatke*.*	Sharrengo (Gedio)	SNNP	Sh	Rt	Or	Crushing fresh root with water and taking that for several days	(Mesfin et al., 2009)
*61*		*Myrsine Africana* L.	Qacama (Oro)	Oro	Sh	Lv		Leaves crushed and squeezed in fresh form with water. The juice was then indicated to be drunk in very small amount for three days	(Gizachew et al., 2013; Yineger and Yewhalaw, 2007; Wolde and Gebre-Mariam, 2002; Desissa and Binggeli, 2000)
*62*	Ranunculaceae	*Clematis hirsute* Perr. & Guill.	Fiitii (Oro)	Oro	C	Lv	Sk/To	Pounding the leaves, dissolving in water &drinking half of small glass & applying certain amount of the solution into the wound's opening using syringe, and also putting residues on its opening (gland TB)	(Fenetahun and Eshetu, 2017; Ashagre et al., 2016; Temam and Dillo, 2016)
*63*		*Clematis simensis* Fres.	Azo-hareg (Amh)	SNNP/Oro	C	AP	Or	Not specified	(Temam and Dillo, 2016; Geyid et al., 2005) (
*64*	Rutaceae	*Citrus limon* (L.) Burm.f.	Lemin (Tig)	Tig	Sh	Fr	Or	Not specified	(Zenebe et al., 2012)
*65*		*Clausena antisata* (Willd.) Benth.	Agam (Amh)	Oro	Sh	Lv	Or	Not specified	(Gizachew et al., 2013; Yineger and Yewhalaw, 2007)
*66*	Rosaceae	*Rosa x richardii* Rehd*.*	Tsigereda	Amh	Sh	Fl	Sk/To	As a skin tie (Gland TB) and also for Bone TB	(Alemneh, 2021a, Alemneh, 2021b)
*67*		*Rubus apetalus* Poir	Go'ra (Oro)	SNNP	Sh	Rt	Or	The root is pounding root, boiling, and drinking	(Tuasha et al., 2018; Gedif and Hahn, 2003)
*68*	Rubiaceae	*Psydrax schimperiana* (A.Rich.) Bridson	Gaalle	Oro	T	Rt		Not specified	(Gemechu et al., 2013; Lulekal et al., 2008)
*69*		*Rubia cordifolia* L.	Mencherer	Amh	C	Rt	Or	Crushing and smashing root in water in 3 days then drink	(Chekole, 2017)
*70*	Agaveace	*Indigofera amorphoides* Jaub. et Spach	Jeere (Oro)	Oro	H	Rt		Not specified	(Gemechu et al., 2013; Lulekal et al., 2008)
*71*	Amaranthaceae	*Celosia polystachia* (Forssk.) C.C. Towns.∗	Kontoma (Af)	Afar	H	Rt	Or, Ins	Root consumption	(Teklehaymanot, 2017)
*72*	Amaryllidaceae	*Scadoxus multiorus* (Martyn) Raf.	Ija Dhukkubsituu (Or)	Amh	H	Rt	Sk/To	Not specified	(Alemneh, 2021a, Alemneh, 2021b)
*73*	Apiaceae	*Anethum graveolens* L. (dill)	*Ensilal (Amh)*	Tig	H	AP	Or	Not specified	(Balcha et al., 2014)
*74*	Araceae	*Arisaema schimperianum* Schott	Amoch (Amh)	Oro	H	Lv	Or	Not specified	(Yineger et al., 2008)
*75*	Asphodelaceae	*Aloe species*	Quureyta (Af)/Riet (Amh)	Afar/Amh	Sh	St/Rt	Or	Drinking its infusion *mixed with roots of Tamarix aphylla and* root of *Salvadora persica L,*. Also, taking orally dried, powdered root buried for 6 months mixed with honey or only *Aloe sp* root buried for 6 months, dried and powdered then mixed with 1kg of honey and taken orally	*(*Zewdu et al., 2015; Seifu, 2004)
*76*	Balanitaceae	*Balanites aegyptiaca* (van Tieghem) Blatter	Uda (Af)	Afar	Sh	Lv	Or, Ins	Fresh leaves consumption	(Teklehaymanot, 2017)
*77*	Boraginaceae	*Bourreria orbicularis* (Hutch. & E.A. Bruce) Thulin	Ulageita (Af)	Afar	Sh	Fr	Or, Ins	Bovine TB (fresh fruit consumption)	(Teklehaymanot, 2017)
*78*	Brassicaceae	*Lepidium sativum* L.	Shunfax (Som)	Som/Oro	H	Sd	Or, Sk/To	Swallowing fresh seeds, applying on open swelling or wound, adding small amount of sulphur & covering it with seed paste of *L. Sativum* & latex of *C. Procera (*EPTB *topical-for gland TB)*	(Temam and Dillo, 2016; Araya et al., 2015; Issa, 2015)
*79*	Canellaceae	*Warburgia ugandensis* Sprague	Kenefa/Zogdom (Amh)	Oro	T	Bk		Not specified	(Giday, 2009a, 2009b; Lulekal et al., 2008; Wube et al., 2005)
*80*	Celastraceae	*Maytenus senegalensis* (Lam.)	Kombolicha (Oro)	Oro	Sh	Rt	Or	Powdered or as an infusion (taken in/drunk)	(Bekele and Reddy, 2014)
*81*	Lauraceae	*Persea americana* Mill	Avocado	Amh	T	Lv		Not specified	(Kahaliw, 2016)
*82*	Logianiaceae	*Buddleja polystachia*	Anfar- (Tig)	Tig	T	Lv	Or	Not specified	(Balcha et al., 2014)
*83*	Loranthaceae	*Tapinanthus globiferus* (A. Rich.) Tiegh.	Hafa-teketsila (Amh)	Amh	H	WP	Sk/To	Appliying on Skin for Gland TB	(Giday et al., 2007)
*84*	Meliantaceae	*Bersama abyssinica* Fresen	Jejjebba	SNNP	Sh	Rt	Or	Crushing/pounding fresh root mixed with cold water and taking orally	(Mesfin et al., 2009)
*85*	Moraceae	*Ficus palmata* Forssk	Qotilebele-s	Tig	Sh	Lv	Ins	Crushing its leaves with roots of *C. Procera* is into powder and mixing with pounded bark of *Croton macrostachyus* &sniffing	(Araya et al., 2015)
*86*	Olacaceae	*Ximenia americana* L.	Hudhaa (Oro)	Oro	T	Rt	Or	Chewing, infusion with hot drinks, eating together with other foods	(Wondimu et al., 2007)
*87*	Plumbaginaceae	*Plumbago zeylanica* L.	Amira (Agew)	Amh	Sh	Lv	Sk/To	Crushed leaves and skin tie (Gland TB) and also for Bone TB	(Giday, 2009a, 2009b; Teklehaymanot, 2009)
*88*	Santalaceae	*Osyris quadripartita* Decn	Waatoo (Oro)	Oro	Sh	Lv, Rt	Or	Pounding them to make solution and drinking 1 water glass daily for a month	(Ashagre et al., 2016)
*89*	Thymelaeaceae	*Gnidia involucrata* Steud	Boto (Amh)	Amh	H	Rt	Or	The root mixed with the fruit of *Cucumis ficifolius* and bulb of garlic are crushed and soaked 7 days in local “Tella” and one cup is taken for five days	(Gebeyehu, 2011)
*90*	Xygophyllaceae	*Balanites aegyptiacus* (L.) Delile	Mekie (Tig)	Tig	T	Fr	Or	Not specified	(Zenebe et al., 2012)
*91*	Polygonaceae	*Rumex abyssinicus* Jacq.	Mekmoko (Oro)	Tig/Oro	H	Rt	Sk/To	Making paste and mixing with cow butter as ointment	(d’Avigdor et al., 2014; Moravec et al., 2014; Zenebe et al., 2012; Gebeyehu, 2011; Abebe et al., 2003; Gedif and Hahn, 2003)
*92*	Salvadoraceae	*Salvadora persica* L	Qadayto (Af)	Afar	T	Rt	Or	Making ihe infusion of the root, and the leaves of *Aloe sp.* And administering orally with root of *Tamarix aphylla*	(Seifu, 2004)
*93*	Sapindaceae	*Dodonaea angustifolia* L.F.	Kitkita (Amh)	Tig/SNNP	Sh	Fr	Or	Powdering dry fruit with water and giving orally	(Balcha et al., 2014; Birhane et al., 2011; Mesfin et al., 2009)
*94*	Scrophulariaceae	*Striga hermonthica* (Del.) Benth	Adiri bereka (Tig	Tig	H	Lv	Or	Crushing the leaf, homogenizing with water anddrinking	(Gidey et al., 2015)
*95*	Tamaricaceae	*Tamarix aphylla* (L.) Karst	Saaganto (Af)	Afar	T	Rt	Or, Ins	Making infusion of its root with root of *Tamarix aphylla* and leaves of *Aloe spp* and administer orally with *Salvadora persica*.	(Seifu, 2004)
*96*	Vitaceae	*Celtis Africana* Burm.f.	Aga (Ku)	Tig	C	WP	Or	Crushing together with bark of *Boscia angustifolia* homogenize with water and drinking a bottle cup of the solution for 7 consecutive days in the morning	(Gidey et al., 2015)
*97*	Viscaceae	*Viscum tuberculatum* A. Rich	Cudurka Qaaxada (Sum)	Som	T	Lv	Or	Grounding leaves, disperse in water & drink	(Issa, 2015)
*98*	Zingiberaceae	*Zingiber offfcinale* Roscoe	Zingibil (Amh)	Amh/Tig	H	Rh	Or	Chewing and swallowing (bone TB)	(Giday et al., 2007)

Key: growth forms (T = tree, B = bulb, Cl = climber, H=Herb, Sh = shrub, Rh= Rhizome).

PU-Parts used = (Lf = leaf, Rt = root, Ba = bark, Fl = flower, Fr = fruit, Sd = seed, Lq = liquid, Sh = shoot, St = stem, AP = Aerial part, WP = Whole part).

Routes of administration = ROA (Or = oral, Sk/To = Skin tie or Topical, Ins = intranasal).

Local names: Amh = Amharic, G = Gurage, Tig = Tigrigna, Oro = Afaan Oromoo, Sid = Sidamu-afoo, Age: Agewugna, Kem = Kambatissa, Som = Somali, Ku = kunama, NA = not available.

Types of TB: EPTB = extrapulmonary TB, BTB = bovine TB.

### 3.2 Growth habit of medicinal plants, parts used, condition of preparations and routes of administration

#### 3.2.1 Growth form of plants used for TB treatment

The growth forms of herbal remedies of TB indicated that the shrubs had the highest proportion with 35.7% of the species while trees (29.6%), herbs (22.4%) and climbers (9.2%) made up the second highest proportion. The remaining 3.1% were the bulbs.

#### 3.2.2 Plant parts used for remedy preparation

Many plant parts are utilized in Ethiopia for anti-TB remedy preparation. Most of the preparation of herbal TB medicines involved the use of a single plant part (95.9%). Plant roots (31.6%) occupied the largest proportion followed by the leaves (28.6%). In a few of TM of TB, use of aerial plant parts (n = 4), seeds (n = 4) and barks (n = 4) were also indicated. But in the remaining proportion, different parts of the plants were mixed together to prepare traditional TB remedies. Flowers, stems and the whole plant parts were reported as very rarely used parts for the preparation. Moreover, majority of the remedies were prepared from freshly harvested parts of medicinal plant species (73.5%) (Table 2).

#### 3.2.3 Preparation and routes of administration of herbal recipes for TB treatment

Different formulations and application procedures of medicinal plant preparations were used to treat TB across the regions of Ethiopia. The most commonly used route of administration was oral (59.2%) followed by dermal/topical route (for gland TB), (10.2%). Intranasal application or sniffing is the least reported route of application, (3.1%). But for (16.7%) plant species the administration routes of TB TM have not been reported. The major modes of remedy preparation from medicinal plant materials were crushing (52%) followed by pounding (29.6%) (Table 2).

Out of a total of all reported traditionally used TB remedies 87.7% and 10.4% plant species were described to be used for the treatment of pulmonary TB (PTB) and extra-pulmonary TB (EPTB), respectively, while 5.2% were used for bovine TB (BTB) (Table 2).

### 3.3 Solvents and additives for preparation of anti-TB herbal medicines

The reported herbal medicines of TB in Ethiopia are prepared by using fresh material, dried form and in some cases either fresh or dried form of the plant parts. During the preparation of most of the TM of TB, water is used as a solvent and in some cases milk and alcohols are added. Milk, cow butter and honey are the commonly used additives to prepare the medicinal plant materials. A few of these TM are also recommended to be taken with hot drinks and “injera” Table 2.

### 3.4 Geographic distribution and frequency of citations of anti-TB medicinal plants

The largest number of herbal TB treatments were reported from Oromia Regional State (n = 22; 22.4%) followed by Tigray (n = 16; 16.3%) and Amhara, (n = 14; 14.3%). From each of the Southern Nations, Nationalities and Peoples Regional (SNNPR) States and Afar region (n = 13; 13.3%) plant species were described. In the study reports across the country, *Croton macrostachyus (n= 7), Allium sativum (n = 5), Myrsine africana (n = 4), Zingiber offfcinale (n = 4)* and *Allium ursinum (n = 4)* are the most frequently reported plant species. The frequency of reports across the regions and distribution in the Ethiopian Flora Region are shown in Table 3.

**Table 3:** The most frequently reported herbal medicines used for the treatment of TB in Ethiopia.

Scientific Name (Family)	Total reports	Areas/regions of reports	References
*Croton macrostachyus*	7	SNNP/Amh/Tig/Addis Ababa	(Balcha et al., 2014; Geyid et al., 2005; Tefera and Kim, 2019; Gonfa et al., 2015; Kewessa et al., 2015; Amsalu, 2010; Gemechu et al., 2013)
*Allium sativum*	5	Oro/Amh/SNNP	(Osman et al., 2020; Belayneh et al., 2012; Wondimu et al., 2007; Mesfin et al., 2009; Birhanu et al., 2015)
*Myrsine africana*	4	Oro/Addis Ababa/Benishangul	(Wolde and Gebre-Mariam, 2002; Desisa, 2000; Yineger and Yewhalaw, 2007; Gizachew et al., 2013)
*Zingiber offfcinale*	4	Amh/Tig	(Teklay et al., 2013; Giday et al., 2007)
*Allium ursinum*	4	Oro/SNNP/Tig	(Balcha et al., 2014; Gemechu et al., 2013; Belayneh et al., 2012; Yirga, 2010)
*Ocimum lamiifolum*	4	Oro/SNNP/Tig	(Getahun, 1976; Gizachew et al., 2013; Mesfin et al., 2009; Mesfin et al., 2005)
*Clematis hirsuta*	3	Oro/SNNP/Tig	(Temam and Dillo, 2016; Fenetahun and Eshetu, 2017; Ashagre et al., 2016)
*Dodonaea angustifolia*	3	SNNP/Tig	(Balcha et al., 2014; Birhane et al., 2011; Mesfin et al., 2009)
*Ekebergia capensis*	3	SNNP	(Tefera and Kim, 2019; Kewessa et al., 2015; Banerjee et al., 2014)

### 3.5 Medicinal plants with documented experimental/clinical evidence for anti-mycobacterial activity

Seventy eight (79.6%) plant species reported in this review had no experimental/clinical evidences for their ability to kill the etiologies of TB. *Allium ursinum, Dodonea anguistifolia* (Balcha et al., 2014; Gemechu et al., 2013)*, Artemisia abyssinica, Croton macrostachys, Eucalyptus camaldulensis, Ocimum basilicum* (Gemechu et al., 2013)*, Otostegia integrifolia* (Kahaliw, 2016; Enyew et al., 2014)*, Pterolobium stellatum* (Balcha et al., 2014)*, Carissa edulis, Persea americana, Vernonia amygdalina* (Kahaliw, 2016) were some of the plants on which clinical/experimental investigations were carried out in Ethiopian research centers and Universities. Though all the remaining plant extracts show the ability to kill mycobacterial species, *Carissa edulis, Vernonia amygdalina* (Kahaliw, 2016) and *Anethum graveolens* (Balcha et al., 2014), failed to show any anti-mycobacterial activities. Particularly, *Otostegia integrifolia* (Kahaliw, 2016; Enyew et al., 2014) *Persea americana* (Kahaliw, 2016), *Pterolobium stellatum* (Kahaliw, 2016; Balcha et al., 2014) and *Jasminum abyssinicum* (Geyid et al., 2005) were reported to show significant anti-mycobacterial activities (Table 4).

**Table 4:** List of medicinal plants with documented experimental/clinical evidence for anti-mycobacterial activity.

Botanical name	Family Name	Parts used	Effectiveness	Solvent/Extraction done by	References
*Allium ursinum*	Alliaceae	Bu	Reported as effective	Methanolic extract-	(Balcha et al., 2014)
*Anethum graveolens*	Apiaceae	AP	Reported as negative	Methanolic extract-	(Balcha et al., 2014)
*Artemisia abyssinica*	Lamiaceae	Lv	Reported as effective	80% methanolic crude extracts	(Gemechu et al., 2013)
*Buddleja polystachia*	Logianiaceae	Lv	Reported as negative	Methanolic extract-	(Balcha et al., 2014)
*Calpurnia aurea*.	Fabaceae	Rt	Reported as effective	80% methanolic crude extracts	(Gemechu et al., 2013) (Zenebe et al., 2012)
*Carissa edulis Vahl*	Apocynaceae	Rt	Failed	Chloroform- maceration	(Kahaliw, 2016)
*Clausena antisata*	Rutaceae	Lv	Reported as effective	Crude aqueous and meoh extracts	(Gizachew et al., 2013; Yineger and Yewhalaw, 2007)
*Dodonea anguistifolia*	Sapindaceae	Lv	Reported as effective	Methanolic extract-	(Balcha et al., 2014)
*Eucalyptus camaldulensis*	Myrtaceae	Lv	Reported as effective	80% Methanolic crude extracts	(Gemechu et al., 2013) (Birhane et al., 2011)
*Jasminum abyssinicum*.	Oleaceae	AP	Reported as effective	Methanol extract- soxhlet	(Geyid et al., 2005)
*Myrsine africana*	Myrsinaceae	Lv	Reported as effective	Crude aqueous and methanolic extracts	(Gizachew et al., 2013; Wolde and Gebre-Mariam, 2002; Desissa and Binggeli, 2000; Yineger and Yewhalaw, 2007)
*Ocimum basilicum*	Lamiaceae	Sd	Reported as effective	80% methanolic crude extracts	(Gemechu et al., 2013)
*Otostegia integrifolia*	Lamiaceae	Rt	Reported as effective with significant Anti-MTB activity	Chloroform- maceration/80% methanol- soxhlet	(Kahaliw, 2016) (Enyew et al., 2014)
*Persea americana*	Lauraceae	Lv	Reported as effective with significant Anti-MTB activity	Acetone/80% methanol	(Kahaliw, 2016)
*Pterolobium stellatum*	Fabaceae	Rt	Reported as effective with significant Anti-mycobacterial activity	Chloroform/80%- maceration methanol- soxhlet	(Kahaliw, 2016; Balcha et al., 2014)
*Vernonia amygdalina.*	Asteraceae	Rt	Failed	Chloroform- maceration	(Kahaliw, 2016)
*Warburgia Ugandensis*	Canellaceae	Ba	Reported as effective with significant Anti-mycobacterial activity		(Giday, 2009a, 2009b; Lulekal et al., 2008; Wube et al., 2005)
*Croton macrostachyus*	Euphorbiaceae	LV	Reported as effective with significant Anti-mycobacterial activity	Methanolic extract-	(Gemechu et al., 2013; Geyid et al., 2005)
*Coccinia abyssinica*	Cucurbitaceae	Rt	Reported as effective its juice has saponin as an active substance and is used to treat TB		(Dawit and Estifanos, 1991)
*Clematis simensis*	Ranunculaceae	AP		Methanolic extract-	(Geyid et al., 2005)

## 4 Discussion

Ethiopia is endowed with abundant medicinal plant resources and traditional herbal practices. Majority of its people live in rural areas and still relies on TMPs for the treatment of human and livestock ailments including TB (Abebe, 2001; Ashagre, 2011; Banerjee et al., 2014; Genene and Hazare, 2017). However, available research evidences on herbal remedies of TB in the country is highly fragmented.

In this review, 98 different plant species from 82 genera and 49 families that are used to treat TB traditionally were retrieved but it was found higher than review reports from India (Arya, 2011), South Africa (Semenya and Maroyi, 2013) and Uganda (Bunalema et al., 2014) that reported 48, 21 and 90 plant species, respectively. Higher report of anti-TB herbal medicines indicates the reliability of Ethiopians on TM, and this could be due to the high cost of modern drugs, paucity and inaccessibility of modern health services, and cultural acceptability of herbal medicines (Agize et al., 2013; Banerjee et al., 2014; Gedif and Hahn, 2003; Teklehaymanot and Giday, 2010; Seifu, 2004). Of these plant species, shrubs had the highest proportion (35.7%) of plant species which are followed by trees (29.6%), and herbs (22.9%). This finding is consistent with a number of ethno-botanical studies from Ethiopia (Bhatcha, 2013; Abebe, 2011; Alemneh, 2021a, Alemneh, 2021b; Jima and Megersa, 2018; Gonfa et al., 2015) and beyond (Obakiro et al., 2020; Bhatcha, 2013). This may be explained by the fact that shrubs are perennial in the arid or sub-arid environments and may be available for use as MPs.

Plants belonging to family Lamiaceae (8 species), Euphorbiaceae (7 species), Cucurbitaceae (6 species) and Fabaceae (6 species) were found as dominant families from which herbal remedies of TB prepared. Moreover, this review's finding of plant species belonging to Lamiaceae, Euphorbiaceae and Fabaceae is in line with the reports of Obakiro et al. from Eastern African countries that included Kenya, South Sudan Tanzania and Uganda (Obakiro et al., 2020; Tabuti et al., 2010). Moreover, significant anti-tubercular activity of plants from family Lamiaceae were also reported from Turkey (Askun et al., 2013) and Nigeria (Ibekwea et al., 2014), implying their higher potential as a target of future study. Moreover, plants belonging to the family Fabaceae were experimented to have biosynthetic phytochemicals with effective anti-mycobacterial activity in Ethiopia and Nigeria (Gemechu et al., 2013; Mann et al., 2008c; Ibekwea et al., 2014). However, plants in Hyacinthaceae, Moraceae and Rutaceae families were the most represented ones in a study from Southern Africa (Semenya and Maroyi, 2013).

According to this systematic review, 22(22.4%) of the herbal TB treatments were reported from Oromia Regional State followed by Tigray 16(16.3 %) and Amhara, 14(14.3%). From each of the SNNPR and Afar regional States, 13(13.3%) plant species were described.

Of the study reports across the country, *Croton macrostachyus, Allium sativum, Myrsine Africana, Zingiber offfcinale* and *Allium ursinum* were the most frequently reported plant species with frequencies of 7, 5, 4, 4, and 4, respectively. Similarly, studies that covered countries of Eastern Africa (Obakiro et al., 2020), India (Gupta et al., 2010; Arya, 2011) and others (Mann et al., 2008c) also revealed the potential of anti-tubercular activities of these plants. Therefore, these plant species should be considered as prime candidates for further in-depth experimental investigations. As the strains of mycobacteria are emerging and changing with specificities in some localities, these plant species could be used to tackle the challenges in TB control (Dawit and Estifanos, 1991; Worku, 2019; Siddiqui et al., 2014).

It is also disclosed that the use of a single plant part (96.9%) of which, the plant roots (31.6%) occupied the largest proportion followed by the leaves (28.6.1%) is more common. Flowers, stems and the whole plant parts were reported as very rarely used parts for the preparation. These findings are also found to be consistent with other studies (Giday et al., 2010; Lulekal et al., 2008) that reported leaves and roots as dominant parts against TB (Arya, 2011; Singh et al., 2015). But the use of plant roots for remedy preparation could significantly affect the sustainability of these herbal medicines unlike the use of aerial parts (Belayneh et al., 2012; Gedif and Hahn, 2003; Moges et al., 2019).

This review has also described oral and intranasal routes (>75%) as the most commonly used routes of administration, implying the herbal remedies are safe for systemic applications, and this was indicated in other studies from Ethiopia (Tesfahuneygn and Gebreegziabher, 2019), Malaysia (Sabran et al., 2016), India (Arya, 2011) and Eastern Africa (Obakiro et al., 2020).

The frequency of reports across the regions and distribution in the Ethiopian Flora are different but available experimental evidences are rare in the country in contrast to a study done in Nigeria (Ibekwea et al., 2014). Seventy eight (79.6%) of the plant species reported in this review had no experimental/clinical evidences for their ability to kill the etiologies of TB. Some evidences on the effectiveness of anti-mycobacterial activities of some herbal remedies of TB were done on *Allium ursinum, Artemisia abyssinica, Carissa edulis, Croton macrostachys, Dodonea anguistifolia, Eucalyptus camaldulensis, Ocimum basilicum, Otostegia integrifolia, Persea americana, Pterolobium stellatu, Vernonia amygdalina*. While there were reports indicating negative anti-mycobacterial activities *of Carissa edulis, Vernonia amygdalina* (Kahaliw, 2016) and *Anethum graveolens* (Balcha et al., 2014). Particularly, *Otostegia integrifolia* (Kahaliw, 2016*;* Enyew et al., 2014) *Persea americana* (Kahaliw, 2016), *Pterolobium stellatum* (*Forsk*)*, Brenan* (Kahaliw, 2016*;* Balcha et al., 2014) and *Jasminum abyssinicum* Hochst (Geyid et al., 2005) were reported to show significant ability to kill mycobacterial species (Table 3). This was also indicated in other studies. Experimental investigations of available anti-TB TMPs are much important for the purpose of potential identification of new antituberculosis drug regimens that further assist standardization of plant-based anti-TB recipes (Bunalema et al., 2014; Ibekwea et al., 2014; Arya, 2011) but in Ethiopia much remains to be done.

## 5 Conclusion

In Ethiopia, TB remains one of the most difficult public health concerns and majority of its people across the country still rely on a number of plants for its treatment. However, majority of these anti-TB plant species used by herbal practioners are not supported with scientific investigation, and this warrants further experimental and clinical validations of these commonly used TMPs of TB. Moreover, the efficacy, toxicity and safety tests should be initiated and this would help in the rapid identification of new anti-TB regimens, and possibly it will lead to a more effective drug development that could help in combating against the rapidly emerging and changing strains of TB etiologies with specificities in some localities.

## Declarations

### Author contribution statement

All authors listed have significantly contributed to the development and the writing of this article.

### Funding statement

This research did not receive any specific grant from funding agencies in the public, commercial, or not-for-profit sectors.

### Data availability statement

Data included in article/supp. material/referenced in article.

### Declaration of interests statement

The authors declare no conflict of interest.

### Additional information

No additional information is available for this paper.
